# *Emx2* lineage tracing reveals antecedent patterns of planar polarity in the mouse inner ear

**DOI:** 10.1242/dev.202425

**Published:** 2024-05-16

**Authors:** Ellison J. Goodrich, Michael R. Deans

**Affiliations:** ^1^ Department of Neurobiology, Spencer Fox Eccles School of Medicine at the University of Utah, Salt Lake City, UT 84112, USA; ^2^ Department of Otolaryngology – Head and Neck Surgery, Spencer Fox Eccles School of Medicine at the University of Utah, Salt Lake City, UT 84132, USA

**Keywords:** Hair cell, Vestibular, Inner ear, Planar polarity, EMX2

## Abstract

The planar polarized organization of hair cells in the vestibular maculae is unique because these sensory organs contain two groups of cells with oppositely oriented stereociliary bundles that meet at a line of polarity reversal (LPR). EMX2 is a transcription factor expressed by one hair cell group that reverses the orientation of their bundles, thereby forming the LPR. We generated *Emx2*-CreERt2 transgenic mice for genetic lineage tracing and demonstrate *Emx2* expression before hair cell specification when the nascent utricle and saccule constitute a continuous prosensory domain. Precursors labeled by *Emx2*-CreERt2 at this stage give rise to hair cells located along one side of the LPR in the mature utricle or saccule, indicating that this boundary is first established in the prosensory domain. Consistent with this, *Emx2*-CreERt2 lineage tracing in *Dreher* mutants, where the utricle and saccule fail to segregate, labels a continuous field of cells along one side of a fused utriculo-saccular-cochlear organ. These observations reveal that LPR positioning is pre-determined in the developing prosensory domain, and that EMX2 expression defines lineages of hair cells with oppositely oriented stereociliary bundles.

## Teaser

**Summary:** EMX2 expression in the otic prosensory domain marks cell lineages corresponding to hair cells of defined stereociliary bundle polarity and restricted to one side of the mouse utricle or saccule.

## INTRODUCTION

**Fig. 1.**
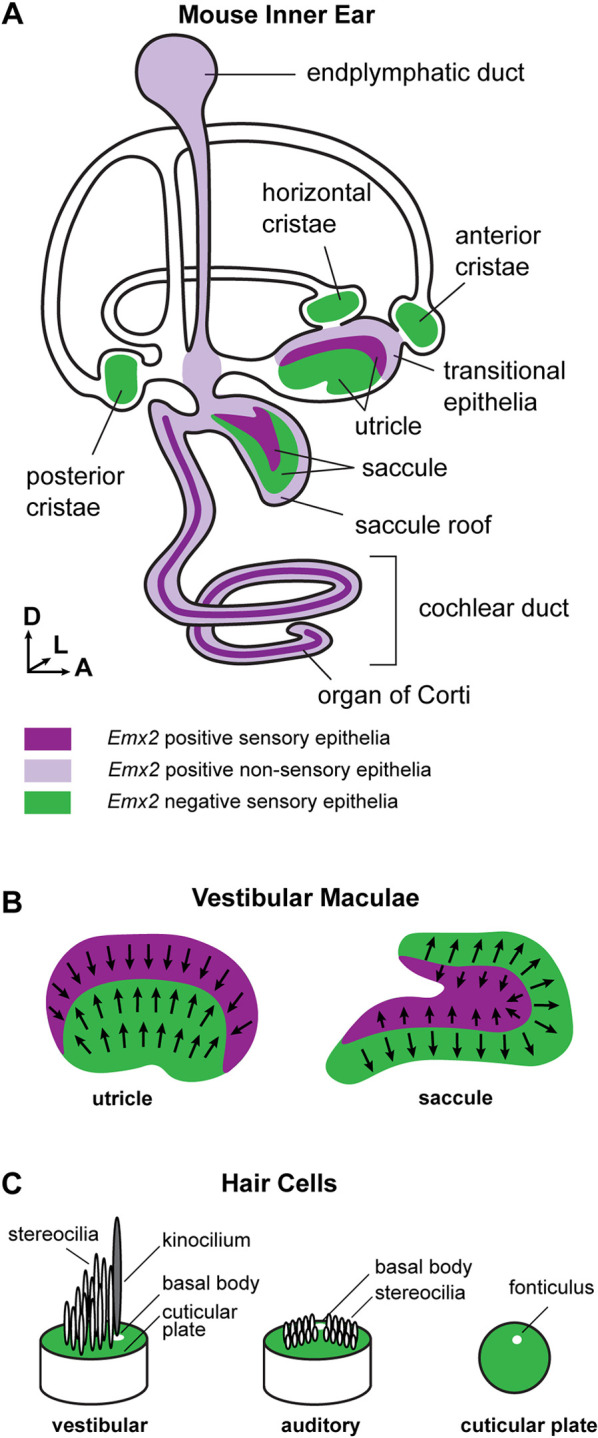
**Patterns of *Emx2* expression are correlated with planar polarity in the mouse inner ear.** (A) Schematic diagram of the membranous labyrinth and sensory epithelia of the mouse inner ear. Magenta shading represents the distribution of *Emx2* expression in sensory (darker shades) and non-sensory (lighter shades) tissue. Green illustrates regions of the sensory epithelia that do not express *Emx2*. (B) The line of polarity reversal (LPR) is positioned in the vestibular maculae at the boundary between *Emx2*-expressing (magenta) and non-expressing (green) regions. In the utricle, hair cells are oriented with the excitatory axis of the bundle pointed towards the LPR, whereas in the saccule the bundles are pointed away from the LPR. (C) The excitation axis of the hair cell is determined by the organization of the stereocilia in rows of increasing height adjacent to a kinocilium that is retained in vestibular hair cells but lost from auditory hair cells at the onset of hearing. In both cell types, a basal body beneath the kinocilium is laterally displaced on the apical cell surface and can be located by the position of the fonticulus.

The mature inner ear contains discrete sensory epithelia that detect sound or motion, and mediate auditory and vestibular function (Fig. 1A). These include two vestibular organs, the utricle and saccule, which respond to linear acceleration and contain mirrored groups of sensory receptor hair cells capable of detecting accelerations oriented in opposite directions (Fig. 1B). Hair cells are specialized mechanoreceptors that extend a bundle of actin-rich stereocilia from their apical surface and transduce microscopic deflections of the stereocilia into neural activity. The stereociliary bundle is structurally and functionally polarized, with the stereocilia arranged in rows of increasing height; only deflections towards the taller stereocilia open mechanically gated ion channels and depolarize the cell (Fig. 1C). A true tubulin-based cilium called the kinocilium is always located adjacent to the tallest row of stereocilia. As a result, the position of the kinocilium and its associated basal body beneath the apical cell surface readily define both the structural and functional polarity of a hair cell.

The stereociliary bundles of utricular and saccular hair cells are embedded in an overlying extracellular matrix called the otoconial membrane, and inertial movements of this membrane during acceleration deflect the stereocilia. The two groups of hair cells in either sensory epithelium have oppositely oriented bundles (Fig. 1B) and, as a result, movements of the otoconial membrane simultaneously excite one group and inhibit the other group of hair cells. The polarized orientation of the bundle is stable because it is anchored in a cuticular plate composed of filamentous actin extending throughout the apical cell surface with the minor exception of a gap, called the fonticulus, that is occupied by the basal body of the kinocilium (Fig. 1C). Head rotation is detected by hair cells in one of three semi-circular canal cristae and sound is detected by hair cells in the organ of Corti of the cochlea (Fig. 1A).

This polarized organization of hair cells is called planar polarity because it occurs parallel to the plane of the sensory epithelium and because the orientation of neighboring cells is coordinated by intercellular signaling mediated by the core planar cell polarity (PCP) proteins (Deans, 2021). In the utricle and saccule, the two groups of hair cells are adjacent and meet at a cell boundary called the line of polarity reversal (LPR). The position of the LPR is determined by expression of the transcription factor EMX2, and hair cells expressing EMX2 always have stereociliary bundle orientations opposite to those that do not (Fig. 1C). Despite this function, EMX2 expression is not restricted to hair cells and occurs throughout sensory and non-sensory structures of the mouse inner ear (Fig. 1A). Sensory structures expressing EMX2 include the auditory hair cells and supporting cells of the organ of Corti in the cochlea, and both hair cells and supporting cells along one side of the LPR in the utricle and saccule. Non-sensory structures expressing EMX2 include the remainder of the cochlear duct, the endolymphatic duct, the transitional epithelia of the utricle and epithelial cells that make up the roof of the saccule. EMX2 is not found in the semi-circular canals or their associated cristae (Fig. 1A). These patterns of expression have been previously shown by endogenous protein and gene expression, and also by genetic labeling with *Emx2*-Cre (Kimura et al., 2005; Holley et al., 2010; Jiang et al., 2017; Stoller et al., 2018; Jia et al., 2023).

EMX2 regulates planar polarity by acting as a master regulator or switch that determines how the stereociliary bundle is oriented relative to an underlying polarity axis established by the core PCP proteins (Deans et al., 2007; Jiang et al., 2017). In *Emx2* mutants, the LPR is lost and all of the stereociliary bundles in the utricle or saccule are oriented in the same direction. Conversely, when *Emx2* is overexpressed in hair cells, bundle orientation is completely reversed and the LPR is similarly absent (Jiang et al., 2017). EMX2 function is similar in the lateral line neuromasts of zebrafish, where *Emx2* is expressed in only half the hair cells, which have the opposite stereociliary bundle orientations to the *Emx2*-negative hair cells (Jiang et al., 2017; Lozano-Ortega et al., 2018; Kozak et al., 2020). Despite these well-characterized effects on hair cell differentiation, *Emx2* expression occurs in the otic vesicle at developmental stages before hair cell specification, and bundle orientation is dependent on the polarity effectors GPR156 (Kindt et al., 2021) and STK32A (Jia et al., 2023), which are expressed exclusively in hair cells and function downstream of EMX2. The early and broad pattern of *Emx2* expression led us to hypothesize that EMX2 pre-determines the position of the LPR by patterning the prosensory domain shortly after its formation in the otic vesicle, and before segregation of the utricle and saccule. This was tested by generating *Emx2*-CreERt2 mice to allow the genetic lineage tracing of cells expressing *Emx2* at early stages of otic development and evaluating their distribution in the mature sensory organs after LPR formation. The potential for the prosensory domain to be patterned by EMX2 was further demonstrated by lineage tracing experiments in the *Dreher* mutant mouse (Nichols et al., 2008; Koo et al., 2009), in which segregation of the utricle and saccule does not occur.

## RESULTS

### Early onset and distribution of *Emx2* expression based upon *Emx2*-Cre genetic labeling

**Fig. 2.**
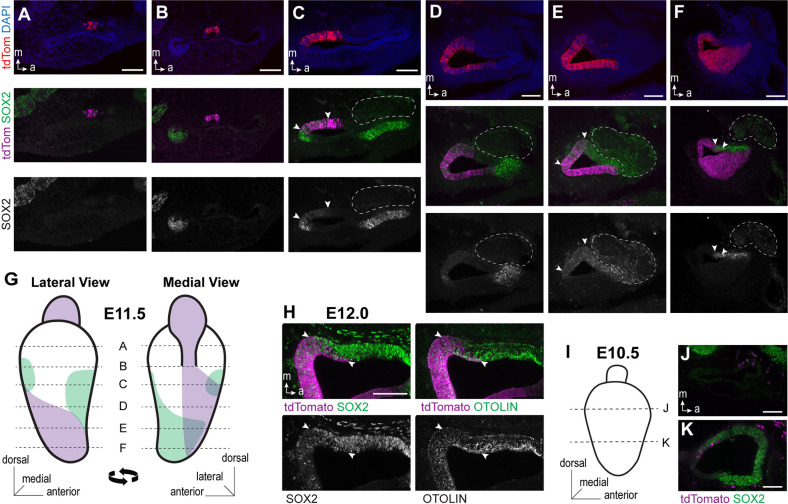
***Emx2*-Cre genetic labeling of the mouse otic vesicle.** (A-F) Representative immunofluorescent images from E11.5 otic vesicle sections demonstrating *Emx2*-Cre-labeled cells (red or magenta) relative to cells expressing SOX2 (green or white; *n*=5 embryos). Arrowheads indicate the boundaries of regions of overlap. (G) Schematic diagram of the E11.5 otic vesicle summarizing the distribution of *Emx2*-Cre labeled cells (purple) and SOX2-expressing (green) cells based upon immunofluorescent labeling of frozen sections. Dashed lines represent the approximate location of fluorescent images in A-F. (H) Immunofluorescent labeling of the otoconial membrane protein otolin reveals a pattern of expression that coincides with the overlapping domains of SOX2 and *Emx2*-Cre tdTomato labeling at E12.0 (*n*=3 embryos). Arrowheads indicate the boundaries of regions of overlap. (I) Schematic diagram of the E10.5 otic vesicle representing the approximate location of images in J and K. (J,K) *Emx2*-Cre genetic labeling initiates in small groups of cells at E10.5 (*n*=9 embryos). Scale bars: 100 µm.

The onset and distribution of *Emx2* gene expression in the developing mouse inner ear can be visualized through genetic labeling by crossing *Emx2*^Cre/WT^ knock-in mice (*Emx2*-Cre; Kimura et al., 2005) with the Cre-dependent reporter ROSA ^tdTom(Ai9)^ (Madisen et al., 2010) to permanently mark *Emx2*-expressing cells with the red fluorescent protein tdTomato. At embryonic day 11.5 (E11.5), *Emx2*^Cre/WT^; *ROSA*^tdTom(Ai9)/WT^ embryos show broad expression of tdTomato in the developing otic vesicle when viewed by immunofluorescent labeling of frozen sections (Fig. 2). At this stage, the otic vesicle has invaginated from the surface ectoderm and neuroblasts destined to form the cochlear-vestibular ganglion are delaminating from the epithelium at the antero-ventral wall of the otic vesicle (Goodrich, 2016). TdTomato can be visualize in all sections along the dorsal to ventral axis of the otic vesicle at this stage. In dorsal sections, expression is restricted to the endolymphatic duct (Fig. 2A,B,G). Starting at the point where the endolymphatic duct emerges from the otic vesicle, TdTomato expands posteriorly along the medial wall and broadens in more ventral sections to include cells from both the medial and lateral sides of the otic vesicle (Fig. 2C-E,G). This expression domain extends into the ventral-most region of the otic vesicle that will form the cochlear duct (Fig. 2F,G). At this stage, genetic labeling includes all regions of the ventral otic vesicle, with the exception of the neurogenic region adjacent to the cochlear-vestibular ganglia.

Regions of the otic vesicle with the potential to become sensory epithelia express SOX2, although at E11.5 the lineage of SOX2-expressing cells also includes non-sensory structures (Gu et al., 2016). Nonetheless SOX2 immunolabeling is a suitable marker for prosensory potential at this stage, and cells genetically labeled by *Emx2*-Cre overlap with SOX2 in two locations. The first is an isolated patch of SOX2-expressing cells on the posterior wall of the otic vesicle that will become the posterior cristae and likely also contributes to the endolymphatic duct (Fig. 2C) (Gu et al., 2016). The second is a region of the medial ventral otic vesicle that extends ventrally through the nascent cochlear duct (Fig. 2E,F). This region overlaps with the SOX2-expressing prosensory domain that will give rise to the utricle, saccule and organ of Corti. Moreover, when evaluated at E12.0, the region of SOX2 and tdTomato overlap includes cells expressing otolin (OTOL1) (Fig. 2H), an extracellular matrix component enriched in the otolithic membrane (Deans et al., 2010). These expression patterns are significant because they suggest that cells destined to reside along one side of the LPR in the lateral region of the utricle or inner region of the saccule already express *Emx2* as early as E11.5. Earlier expression of tdTomato at E10.5 is limited to sparsely distributed cells that do not form a continuous domain (Fig. 2I-K). The onset of *Emx2*-Cre recombinase activity at E10.5 has also been observed with a second Cre-dependent reporter line (Ono et al., 2014), where reporter expression was similarly dispersed. Overall, the onset of robust genetic labeling by *Emx2*-Cre at E11.5 coincided with the appearance of the endolymphatic duct, and included both prosensory and non-sensory regions of the otic vesicle.

### Design and characterization of *Emx2*-CreERt2 mice

**Fig. 3.**
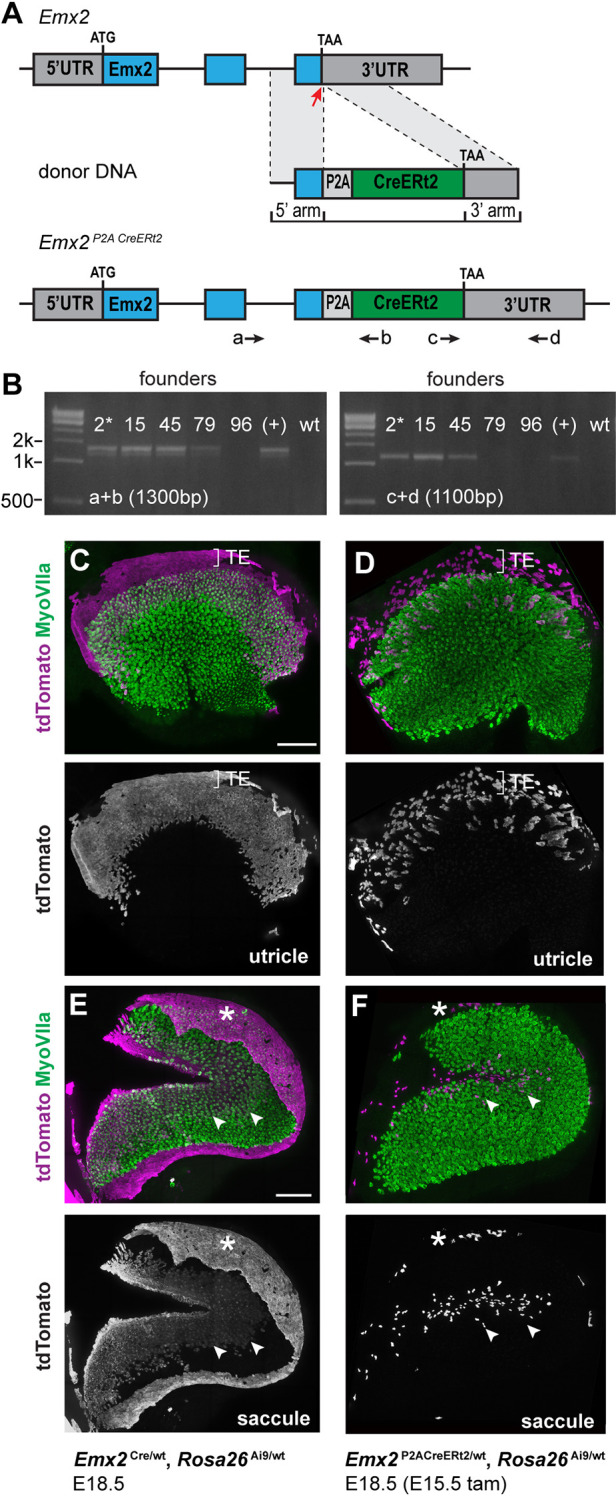
***Emx2*-CreERt2 gene targeting and validation.** (A) A CRISPR-mediated gene-targeting strategy used to generate the *Emx2*-CreERt2 line of transgenic mice. Blue indicates the *Emx2*-coding sequence and the red arrow indicates the position of the Cas9 cut site. (B) PCR-based evaluation of homologous recombination in candidate founders. Positions of PCR primers flanking the 5′ and 3′ homologous arms are illustrated in A (a-d). Mouse 2 (asterisk) was selected to establish the *Emx2*-CreERt2 line. (C,D) *Emx2*-Cre lineage-traced utricle at E18.5 (C) and *Emx2*-CreERt2 lineage traced utricle at E18.5 (D) after activation by a single dose of tamoxifen delivered at E15.5 (*n*=3 utricles/genotype). Brackets indicate the boundaries of the transitional epithelia (TE). (E,F) *Emx2*-Cre lineage-traced saccule at E18.5 (E) and *Emx2*-CreErt2 lineage-traced saccule at E18.5 (F) after activation by a single dose of tamoxifen delivered at E15.5 (*n*=3 saccules/genotype). Asterisks indicate labeled cells in the saccular roof where this tissue was not removed during dissection. Arrowheads indicate the boundary of labeled cells within the saccular sensory epithelium. Scale bars: 100 µm.

To visualize the lineage of cells expressing *Emx2* at E11.5 and determine their distribution relative to the LPR in the mature utricle and saccule, a transgenic mouse expressing tamoxifen-inducible CreERT2 from the endogenous *Emx2* promoter was generated using CRISPR-based gene-editing techniques (Fig. 3A). The targeting strategy generated a bicistronic *Emx2* mRNA by inserting a P2A sequence between the *Emx2* and the transgenic *CreERt2*-coding sequence in order to maintain endogenous EMX2 function. A CRISPR guide RNA was designed to direct a single Cas9 DNA cleavage near the *Emx2* translational stop codon, and donor DNA containing the P2A sequence followed by *CreERt2* replaced the stop codon after DNA repair by homologous recombination. This gene-editing approach did not disrupt the *Emx2*-coding sequence or gene regulatory sequences, or the 5′ and 3′ untranslated regions (UTRs) of the *Emx2*-*CreERt2* bicistronic mRNA. Founder mice were screened by PCR genotyping using PCR primer pairs flanking the 5′ and 3′ homology regions of the donor DNA (Fig. 2B) and a single founder mouse was selected to establish the transgenic line.

Lineage tracing using the *Emx2*^Cre^ knock-in line provides a cumulative readout of *Emx2* transcription during the course of inner ear development when crossed with the tdTomato reporter line Ai9 and evaluated at perinatal stages (Kimura et al., 2005; Madisen et al., 2010; Jiang et al., 2017; Stoller et al., 2018). Within the utricle and the saccule, this pattern includes regions of the sensory epithelia located along one side of the LPR (Fig. 3C,E). Also labeled are non-sensory epithelia located adjacent to the vestibular hair cells. These are the transitional epithelial cells of the utricle (Fig. 3C) and epithelial cells that make up the roof of the saccule (Fig. 3E). A similar pattern of genetic labeling is seen in *Emx2*^P2ACreERt2/wt^; *ROSA*^Ai9/wt^ mice after tamoxifen-induced activation of CreERt2 at E15.5 (Fig. 3D,F). In contrast to Cre recombinase, genetic labeling by CreERt2 marks only cells expressing the transgene for a short period of time after tamoxifen induction, which is estimated to be 24 h, and the number of cells labeled depends upon tamoxifen dose and levels of transgene expression. The lower number of cells labeled by *Emx2*-CreERt2 compared with *Emx2*-Cre likely reflects the levels of CreERt2 expression from the *Emx2* locus, as the number of labeled cells is increased in *Emx2*^P2ACreERt2/P2ACreERt2^ embryos treated with the same amount of tamoxifen (see Fig. 5E,F). Larger doses of tamoxifen proved detrimental to the developing litters, which were frequently miscarried despite the inclusion of progesterone to mitigate this negative effect of tamoxifen treatment.

**Fig. 4.**
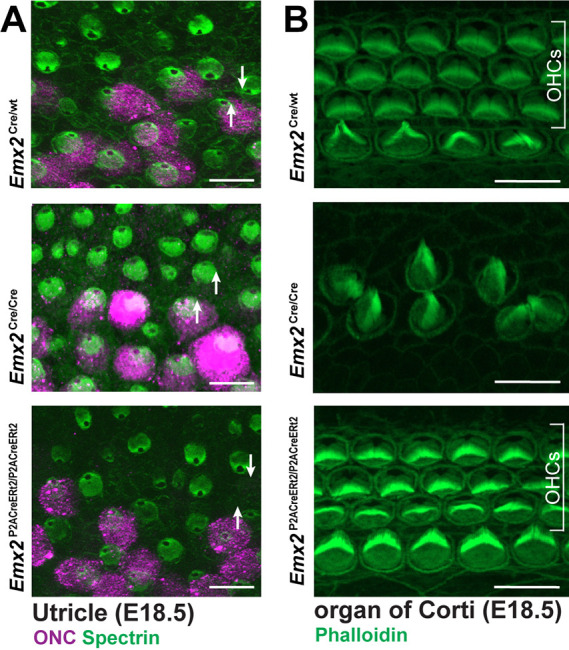
**The *Emx2*-CreERt2 transgene does not disrupt EMX2 function.** (A) The organization of vestibular hair cells and patterning about the line of polarity reversal (LPR) in the utricle of heterozygous control, *Emx2*-Cre and *Emx2*-CreERt2 transgenic mice when the transgenes are homozygosed. βII-Spectrin labels the hair cells (green) and reveals bundle orientation based upon the position of the fonticulus. Oncomodulin (ONC) labels hair cells in the striolar region (magenta), which is located along one side of the LPR. Arrows indicate bundle orientation for representative pairs of hair cells (*n*=3 utricles/genotype). (B) The organization of auditory hair cell stereociliary bundles in the organ of Corti of heterozygous control, *Emx2*-Cre and *Emx2*-CreERt2 transgenic mice when the transgenes are homozygosed (*n*=3 cochlea/genotype). OHCs, outer hair cells. Scale bars: 10 µm.

A consequence of the knock-in strategy employed to generate the *Emx2*-Cre line is that *Emx2*^Cre/Cre^ mice have mutant phenotypes similar to those reported for other *Emx2* mutants (Kimura et al., 2005; Holley et al., 2010; Jia et al., 2023). In these mice, the LPR does not form because vestibular hair cells in the lateral region of the utricle are misoriented and share the same bundle orientations as hair cells in the medial region. In contrast, LPR formation occurs normally in the *Emx2* ^P2ACreERt2/P2ACreERt2^ utricle (Fig. 4A). *Emx2* is also required for outer hair cell (OHC) development in the mouse cochlea (Holley et al., 2010) and OHCs are similarly missing; in addition, the patterning and planar polarity of inner hair cells (IHCs) is disrupted in *Emx2* ^Cre/Cre^ mice (Fig. 4B). In contrast, all three rows of OHCs and the IHCs are intact and organized with normal planar polarity in *Emx2* ^P2ACreERt2/P2ACreERt2^ cochlea (Fig. 4). Together, these phenotypes demonstrate that *Emx2* expression is not disrupted in the *Emx2*-CreERt2 line, in which levels of EMX2 are sufficient to support normal inner ear development.

### *Emx2* lineage tracing throughout inner ear development

The sensory epithelia of the inner ear are derived from a common prosensory domain that is specified within the otic vesicle shortly after invagination and fusion at E10 (Mann et al., 2017; Zak and Daudet, 2021). At this stage, the otic vesicle is also patterned about its major axes by morphogen gradients emanating from peri-otic tissues (Bok et al., 2007a,b; Whitfield and Hammond, 2007). Based upon these patterns, the prosensory domain is segregating into distinct auditory and vestibular domains, and subsequently into separate sensory organ precursors. The first to appear is the posterior cristae followed shortly after by the remaining four vestibular sensory epithelia and the organ of Corti (Morsli et al., 1998). As a result, the utricle, saccule, and anterior and horizontal cristae share a common origin, while the posterior cristae is independently specified. The early expression of *Emx2*-Cre (Fig. 2) suggests that the prosensory region might also be pre-patterned into sub-domains that determine the position of the LPR along their boundaries at later stages. This hypothesis was tested by lineage tracing cells within the developing ear using the *Emx2*-CreERT2 line.

**Fig. 5.**
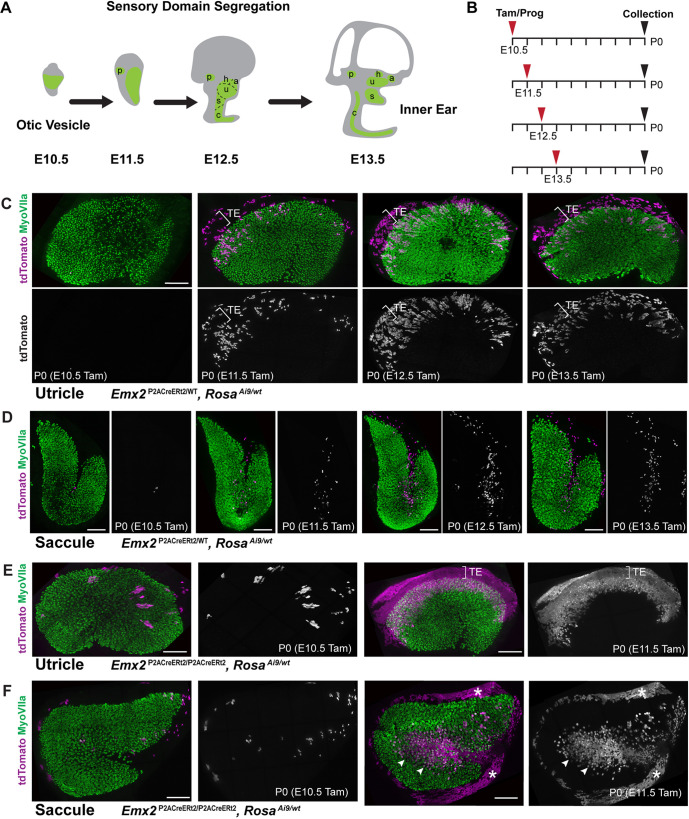
**The *Emx2*-CreERt2 lineage is established before segregation of the vestibular maculae.** (A) Schematic illustrating sensory domain segregation in the mouse inner ear. The sensory epithelia are derived from a common prosensory domain (green) that is segregated over time to form the posterior (p), horizontal (h) and anterior (a) semi-circular canal cristae, the utricle (u) and saccule (s), and the organ of Corti (c). (B) Experimental timeline for *Emx2*-CreERt2 lineage-tracing experiments illustrated in C-F. (C,D) tdTomato expression in P0 *Emx2*^P2ACreERt2/wt^; Rosa^Ai9/wt^ utricles (C) and saccules (D) after a single dose of tamoxifen delivered at E10.5 (*n*=4 utricles, 3 saccules), E11.5 (*n*=6 utricles, 3 saccules), E12.5 (*n*=9 utricles, 9 saccules) or E13.5 (*n*=4 utricles, 4 saccules). Hair cell labeling with MyoVIIa antibodies (green) defines the boundaries of the sensory epithelia; bracket indicates the approximate width of the transitional epithelia. (E,F) tdTomato expression in P0 Emx2^P2ACreERt2/ P2ACreERt2^; Rosa^Ai9/wt^ utricles (E) and saccules (F) after a single dose of tamoxifen delivered at E10.5 (*n*=4 utricles, 4 saccules) or E11.5 (*n*=6 utricles, 6 saccules). Arrowheads indicate the boundary of the line of polarity reversal in the saccule and asterisks indicate labeling of the saccular roof. Scale bars: 100 µm.

To facilitate genetic lineage tracing, *Emx2*^P2ACreERt2/wt^ males were crossed with *ROSA*^Ai9/Ai9^ females, a single dose of tamoxifen supplemented with progesterone was delivered by gavage to timed pregnant mice at E10.5, E11.5, E12.5 or E13.5, and the extent of Cre-mediated lineage tracing was evaluated at P0 (Fig. 5B). For these experiments, it is presumed that CreERt2 is activated by tamoxifen for a 24 h window. Consistent with frozen sections of *Emx2*^Cre/wt^; *ROSA*^Ai9/wt^ otic vesicles at E10.5, tamoxifen induction of *Emx2*-CreERt2 at E10.5 did not label cells within the utricle and saccule when evaluated at P0 (Fig. 5C,D). However, a single dose of tamoxifen delivered at E11.5 was sufficient to label cells within the sensory epithelia of the utricle and the adjacent transitional epithelia (Fig. 5C). Sparse labeling of cells within the inner region of the saccule was also seen at this stage (Fig. 5D). Labeling of both the utricle and saccule was more robust when tamoxifen was delivered at E12.5, and was consistently seen after induction at later stages (Fig. 3D,F and Fig. 5C,D). For more robust genetic labeling, the *Emx2*-CreERt2 allele was homozygosed and the lineage of *Emx2*-expressing cells was evaluated at P0 after tamoxifen induction at E10.5 or E11.5 in *Emx2*^P2ACreERt2/P2ACreERt2^; *Rosa*^Ai9/wt^ embryos. Patterns of labeled cells were consistent with the onset of *Emx2* expression and occurred in a subset of cells labeled at E10.5 and throughout the sensory cells located along one side of the LPR in both the utricle and saccule when cells were labeled at E11.5 (Fig. 5E,F). This labeling protocol also marked non-sensory cells in the transitional epithelia and saccule roof in a pattern similar to the cumulative labeling seen with *Emx2*-Cre.

**Fig. 6.**
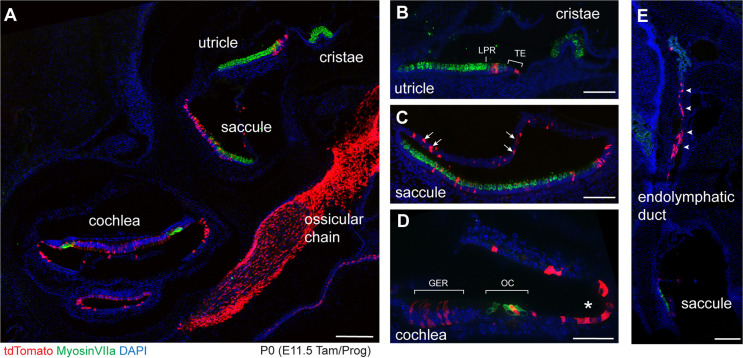
**The *Emx2*-CreERt2 lineage after tamoxifen induction at E11.5.** (A) Cross-section through a *Emx2*^P2ACreERt2/wt^; Rosa^Ai9/wt^ inner ear at P0 that had received a single dose of tamoxifen at E11.5 (*n*=3 mice). Reporter expression is evident in the utricle, saccule and cochlea, in addition to robust labeling of the ossicular chain. No labeling is seen in the cristae. (B) Cross-section of the utricle showing tdTomato-labeled cells along one side of the presumptive line of polarity reversal and the adjacent transitional epithelia (TE). (C) Cross-section of the saccule showing tdTomato-labeled cells within the sensory epithelia as well as non-sensory cells located in the epithelia that make up the roof of the saccule (arrows). (D) A cross-section of one turn of the cochlea showing labeled cells within the greater epithelial ridge (GER) and organ of Corti (OC), and non-sensory cells within the lateral wall of the cochlear duct (asterisk). (E) Labeled cells are also detected within the endolymphatic duct (arrowheads). Scale bars: 200 µm in A,E; 100 µm in B,C; 50 µm in D.

The distribution of sensory and non-sensory cells labeled by *Emx2*-CreERt2 was further evaluated by immunofluorescent labeling of frozen sections of P0 ears after tamoxifen induction at E11.5 (Fig. 6A). Consistent with whole-mount imaging, tdTomato was seen in hair cells and supporting cells of the lateral region of the utricle and transitional epithelia cells (Fig. 6B), in hair cells and supporting cells within the saccule, and in non-sensory cells throughout the roof of the saccule (Fig. 6C). tdTomato was also present throughout the cochlear duct, including the greater epithelial ridge (GER), hair cells within the organ of Corti and cells throughout the lateral wall (Fig. 6D), in addition to the endolymphatic duct (Fig. 6E). Outside of the inner ear, *Emx2*-CreERt2 lineage tracing also revealed *Emx2* expression in cells contributing to the ossicular chain (Fig. 6A), which is consistent with the role of this transcription factor in their development, and the hearing loss reported in the *Emx2* mutant mouse *Pardon* resulting from ossicular malformations (Rhodes et al., 2003).

**Fig. 7.**
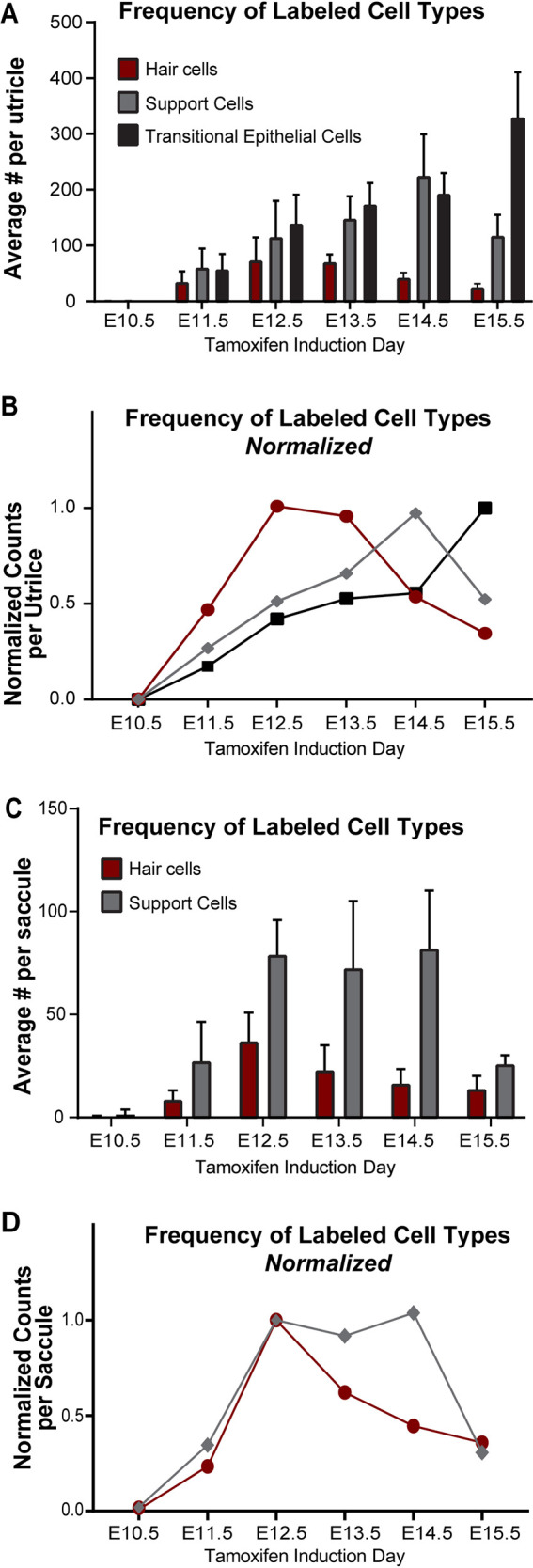
**The frequency of labeled cell types is dynamic and changes across development stages.** (A,B) The average number of cells of each type labeled in the *Emx2*^P2ACreERt2/wt^ utricle at P0 after tamoxifen induction at a single developmental stage (A), and the relative number of cells of each type normalized to the developmental stage of maximum labeling (B) (*n*=6 at E11.5, *n*=9 at E12.5, *n*=5 at E13.5, *n*=5 at E14.5, *n*=8 at E15.5). (C,D) The average number of hair cells and supporting cells labeled in the *Emx2*^P2ACreERt2/wt^ saccule at P0 after tamoxifen induction at a single developmental stage (C), and the relative number of cells of each type normalized to the developmental stage of maximum labeling (D) (*n*=3 at 11.5, *n*=9 at E12.5, *n*=4 at E13.5, *n*=4 at E14.5, *n*=6 at E15.5).

Although the efficiency of Cre-mediated recombination in *Emx2*-CreERt2 mice was low, this level of genetic labeling does enable easy quantification of the frequency distribution of cell types that were labeled at each developmental stage (Fig. 7A,B). Within the utricle, tamoxifen induction at E11.5 resulted in the labelling of a comparable number of hair cells, supporting cells and transitional epithelial cells. However, the frequency of hair cell labeling peaked at E12.5, and at subsequent stages tamoxifen induction was more likely to result in the labeling of supporting cells and transitional epithelial cells. Finally, at E15.5, lineage tracing of transitional epithelial cells surpassed that of hair cells and supporting cells within the sensory epithelia. Although it lacks transitional epithelia, a similar transition occurs in the developing saccule with more-frequent hair cell labeling after tamoxifen administration at E11.5 followed by a preferential labeling of supporting cells at later ages (Fig. 7C,D). As the efficiency of genetic labeling in response to tamoxifen is dependent upon the level of CreERt2 recombinase that is expressed, this progression in cell type labeling frequency may reflect dynamic changes in the rates of *Emx2* transcription between hair cells and non-hair cells during their development. It is also possible that the frequency of supporting cells and transitional epithelial cells labeling increases due to proliferation. Regardless of the underlying mechanism, these recombination profiles will be important to consider if the *Emx2*-CreERt2 line is to be used for cell type-specific gene deletion.

### *Emx2* expression in the otic prosensory domain precedes LPR formation in the vestibular maculae

**Fig. 8.**
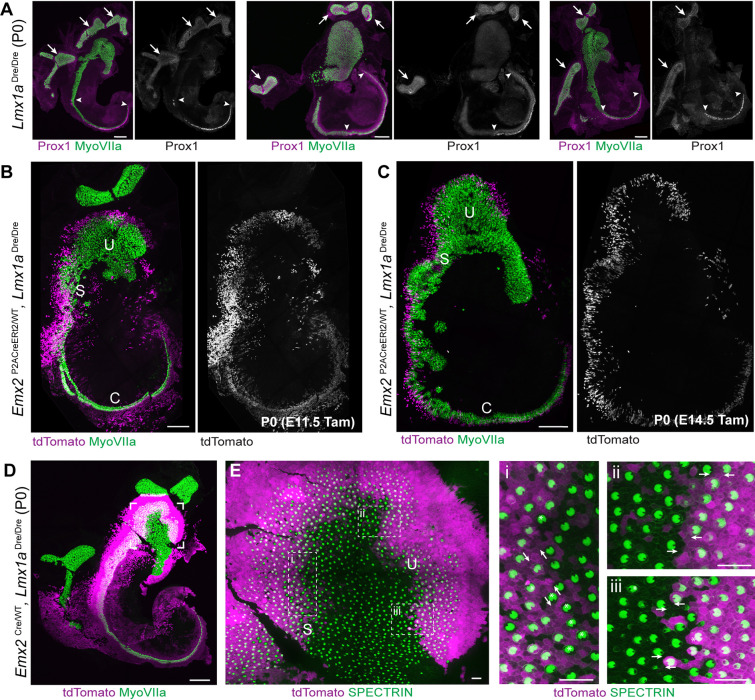
***Emx2*-CreERt2 lineage tracing in *Dreher* mutants.** (A) Three examples of the *Dreher* mutant phenotype in which the utricle, saccule and cochlea fail to segregate and form a continuous field of hair cells (*n*=3 embryos). The transcription factor Prox1 marks auditory hair cells and distinguishes them from hair cells in the presumptive saccule and utricle. Auditory hair cells are bounded by arrowheads. Prox1 also marks vestibular hair cells in the cristae (arrows), the morphogenesis of which is highly variable between individual mutants. (B) *Emx2*-CreERt2 lineage tracing in a *Dreher* mutant at P0 after tamoxifen induction at E11.5 labels a field of cells spanning the presumptive utricle (U) and saccule (S) (*n*=9 mice). (C) *Emx2*-CreERt2 lineage tracing in a *Dreher* mutant at P0 after tamoxifen induction at E14.5 reveals a similar field of labeled cells spanning the fused utricle and saccule (*n*=3 mice). (D) *Emx2*-Cre lineage tracing in a *Dreher* mutant at P0 (*n*=4 mice). (E) Higher magnification image of the outlined region in D showing the boundaries of *Emx2*-Cre labeling in the presumptive utricular-saccular domain, and the orientation of stereociliary bundles at the presumptive saccular (i) and utricular boundaries (ii and iii). Arrows define bundle orientation for adjacent cells; asterisks (i and iii) indicate cells in which orientation and EMX2 identity do not correspond. Scale bars: 200 µm in A-D; 20 µm in E.

One striking line of evidence that the utricle, saccule and organ of Corti are derived from a common prosensory domain is the phenotype seen in mutant mice that affects the transcription factor LMX1A, including the *Dreher*, *Mutanlallemand*, and *Belly Spot and Deafness* lines (Nichols et al., 2008; Koo et al., 2009; Steffes et al., 2012). In *Dreher* mutants, the non-sensory cells forming structures that separate the individual sensory epithelia fail to be specified or to proliferate, resulting in a continuous utriculo-saccular-cochlear organ in which presumptive macular and cochlear organs are contiguous. Development of the semi-circular canal cristae is also disrupted in the *Dreher* mutants, but instead of being fused with the maculae, they form extensive large and branched sensory epithelia. These aberrant cristae are easily identified based on Prox1 expression, and their organization is highly variable between individual mice. In contras,t features of the fused utriculo-saccular-cochlear organ are more consistent between individual *Dreher* mutants (Fig. 8).

The early expression of *Emx2* suggests that EMX2 could pre-pattern the prosensory domain before sensory patch segregation. As *Emx2*-CreERt2 induction at E11.5 marks hair cells in the mature utricle and saccule, *Emx2*-CreERt2 lineage tracing of *Dreher* mutants at this stage should reveal the antecedent pattern of *Emx2* expression, and a continuous field of tdTomato-labeled cells is expected in the *Dreher* mutants. This possibility was tested by intercrossing the *Emx2*-CreERt2 and *Dreher* lines and administering tamoxifen at E11.5. Consistent with this hypothesis, tamoxifen induction at this stage results in a single and continuous field of tdTomato-labeling in *Dreher* mutants when evaluated at P0 (Fig. 8B). This field extends along the boundary of the presumptive utricle and saccule, and into the mutant cochlea. Induction at E14.5, the stage at which the LPR would be forming in wild-type mice (Denman-Johnson and Forge, 1999), results in a similar distribution of tdTomato-labeled cells in the *Dreher* mutant ear (Fig. 8C). The boundary of EMX2 expression is more precisely marked when *Dreher* mutants are lineage traced by *Emx2*-Cre (Fig. 8D), and the orientation of individual hair cells can be evaluated relative to the boundary of tdTomato expression (Fig. 8E). For the presumptive utricular region, bundle orientation resembles a wild-type utricle with hair cells pointed towards the LPR. In the presumptive saccule, hair cells are more likely to be oriented in an anti-parallel manner at the LPR rather than pointed away, as the wild-type saccule. However, the wild-type saccule also differs from the utricle in that hair cells of either orientation are often interspersed at the LPR and a minority of cells share the orientation of EMX2-expressing cells while not being labeled by *Emx2*-Cre (Jiang et al., 2017; Stoller et al., 2018). These characteristics are also evident in the presumptive saccular region of the *Dreher* mutant (Fig. 8E). Based upon these cumulative observations, it appears evident that the lineage of cells expressing EMX2 is established at the earliest stages of prosensory domain development, and that the origin of the LPR is determined before the formation of independent and structurally distinct vestibular maculae.

## DISCUSSION

Using CreERt2-mediated genetic lineage tracing, we demonstrate that the planar polarity transcription factor EMX2 is expressed in the prosensory domain of the developing inner ear before hair cell specification and before segregation of the utricle and saccule into discrete sensory organs. In addition, we show that the lineage of *Emx2*-expressing cells from this stage is restricted to one side of the LPR in the mature sensory organ. Together, these data support the hypothesis that planar polarity signals are established at early stages of inner ear development, where they are pre-positioned to guide subsequent steps of hair cell differentiation, including stereociliary bundle polarization. These observations were made using a new mouse line in which CreERt2 is expressed and regulated by endogenous *Emx2* gene regulatory elements. Based upon patterns of Cre-mediated genetic labeling, the expression of *Emx2*-CreERt2 matches *Emx2*-Cre and patterns of *Emx2* expression determined by immunolabeling and *in situ* hybridization (Holley et al., 2010; Jiang et al., 2017; Stoller et al., 2018; Jia et al., 2023). However, unlike *Emx2*-Cre, the *Emx2*-CreERt2 line does not disrupt EMX2 function, as demonstrated by the lack of mutant phenotypes when the transgene is homozygosed. An unfortunate deficit of *Emx2*-CreERt2 is the limited efficiency of Cre-mediated recombination when tamoxifen is delivered by gavage, as that hinders its usefulness for generating conditional knockout mice. We currently have not determined whether *Emx2*-CreERt2 activation is more responsive to intraperitoneal (IP) delivery of 4-hydroxy tamoxifen or whether CreERt2 induction is more efficient in other tissues where expression from the *Emx2* promoter might be stronger.

### Patterning the developing prosensory domain

The utricle and saccule are derived from adjacent regions of the prosensory domain that have not segregated at E11.5 when *Emx2*-CreERt2 lineage tracing marks cells in both sensory epithelia. Moreover, the early pattern of *Emx2* expression overlaps with the prosensory domain marker SOX2. These results indicate that the prosensory domain is already being functionally patterned in the developing otocyst and that patterning does more than distinguish auditory from vestibular and maculae from cristae, including pre-determining the functionality of the future sensory epithelia. Fritzsch and colleagues had suggested that this may be the case when they proposed that the cochlea shared functional lineage with regions of the utricle and saccule, based upon evolutionary observations (Fritzsch et al., 2002). We propose that these lineages are distinguished by expression of *Emx2* and that the antecedent patterning of the prosensory domain is reflected in the *Emx2*-CreERt2 lineage of *Dreher* mutants (Fig. 8). In this scenario, the *Emx2*-expressing domain that overlaps with SOX2 in the ventro-medial otic vesicle (Fig. 2) contributes to the utricle and saccule, and the boundary of *Emx2* expression at this stage is the nascent LPR. Although our results show that future stereociliary bundle orientations are specified by EMX2 expression at this stage, it is not clear when during the course of differentiation an individual hair cell becomes committed to developing or maintaining a stereociliary bundle with a specific orientation.

A limitation to the current study is that it consists of snapshots from single developmental stages and cannot track the dynamic morphogenesis linking *Emx2*-Cre and SOX2 overlap at E11.5 with EMX2 expression in the mature utricle and saccule. Although imaging these processes in the living embryo is not possible, a detailed timecourse may be practical to construct using light sheet microscopy and 3D renderings of the inner ear if they are collected across a range of developmental stages. Not only would this morphological progression reveal how the saccule is derived from the otic vesicle, but it also has the potential to explain how a boundary of *Emx2* expression in the E11.5 otic vesicle could form LPRs in the utricle and saccule with opposite organizations of stereociliary bundles. It is also likely that morphogenesis of the saccule contributes to the final orientation of stereociliary bundles along this LPR because it is the presumptive saccular region of *Dreher* mutants and the bundles were more often oriented anti-parallel to one another rather than pointing away from an LPR (Fig. 8E). *Emx2*-Cre labeling also did not precisely predict the position of the presumptive LPR in the saccular region of *Dreher* mutants, and cells with differing orientations were more likely to be interspersed than at the utricle boundary. These observations were not unexpected, as Jiang et al. and others similarly reported deviations between *Emx2*-Cre lineage tracing and the position of the LPR in the saccule of control mice (Jiang et al., 2017; Stoller et al., 2018). Nonetheless, these observations suggest that EMX2 may not be the only factor impacting LPR formation in the saccule, and that dynamic processes, including cellular migration and rearrangement, also contribute.

An outstanding question is what determines the boundaries of EMX2 expression at these early stages of otic development, as this pattern is correlated with the position of the LPR in the mature maculae. The only region of the ventral otic vesicle that is not genetically labeled by *Emx2*-Cre at E11.5 is the antero-medial segment adjacent to the cochleovestibular ganglia (Fig. 2). This corresponds to the neurogenic domain from which neuroblasts actively delaminate to form the neighboring ganglia. One possibility is that the neurogenic progression occurring in this domain is incompatible with *Emx2* and the gene is actively repressed. It is also possible that a morphogenic signal released from the newly formed ganglia contributes to otic vesicle patterning by repressing *Emx2* expression in this adjacent region. In either event, this repression appears to be incomplete because a few spiral ganglion neurons are labeled by *Emx2*-Cre (Ghimire et al., 2018), which could be neuroblasts that transiently expressed *Emx2* before delaminating. Alternatively, the boundaries of *Emx2* expression are determined by the combination of morphogenic gradients that are known to establish the primary axes of the otic vesicle at this stage (Bok et al., 2007a). As boundaries of EMX2 expression are associated with the position of the LPR in the mature utricle and saccule, the molecular mechanism(s) that prevent *Emx2* expression in the neurogenic domain of the otic vesicle may have a significant impact on vestibular planar polarity in the mature maculae, and therefore would be important to identify.

It has also been shown that the expression of the core PCP proteins that coordinate the orientation of stereociliary bundles between neighboring hair cells is similarly initiated at these early stages of otic development and is maintained through the course of inner ear morphogenesis (Yang et al., 2017). Based upon similar ideas, we had proposed a model in which a restricted pattern of *Emx2* expression and a uniform field of PCP signaling could organize the utricle and the saccule before their segregation and account for the complementary patterning around their LPRs. In this theoretical model, an annular distribution of EMX2, if divided properly during segregation, would account for the opposite organization of hair cells at the LPR in both maculae (Deans, 2013). Of course, the distribution of *Emx2* lineage tracing we see in *Dreher* mutants is not annular, and the final organization of hair cells in the utricle and saccule is dependent upon morphogenesis that does not occur in *Dreher* mutants. Nonetheless, if these two essential organizing principles were established early enough during development, this would only need to occur once and not independently within each sensory organ. If planar polarity patterns were established early in development, this may also account for how different species have evolved alternative sensory structures that share conserved planar polarity features, including the coordinated orientation of stereociliary bundles and LPRs.

### Does EMX2 strictly regulate regional patterning?

Within the inner ear and lateral line, EMX2 is been well described as a genetic switch that regulates stereociliary bundle polarity (Jiang et al., 2017; Ji et al., 2018; Kozak et al., 2020). Thus, when Emx2 is selectively deleted from hair cells, their bundles are reoriented (Ji et al., 2022), or when Emx2 is selectively overexpressed in hair cells, the LPR is lost because all bundles have the same orientation (Jiang et al., 2017). We have shown that EMX2 functions by negatively regulating the kinase gene *Stk32a*, thereby restricting STK32A expression to hair cells in the medial region of the utricle and outer region of the saccule. Through its kinase activity, STK32A negatively regulates the localization of the planar polarity receptor GPR156 at apical cell boundaries. Owing to this double-negative regulatory circuit, GPR156 can only reorient stereociliary bundles relative to the underlying PCP axis in cells expressing EMX2 (Kindt et al., 2021; Jia et al., 2023).

Despite this recent progress, EMX2 remains better known as a transcription factor that regulates regional identity in the developing neocortex (Bishop et al., 2000; Mallamaci et al., 2000). In the absence of *Emx2*, the size of primary cortical regions changes with reductions in rostral areas and corresponding increases in caudal areas, whereas upon overexpression of *Emx2* these trends are reversed. This process requires crosstalk between mutually antagonistic transcription factors and is a process known as arealization (Bishop et al., 2000; Hamasaki et al., 2004). Within the mammalian inner ear, other features of the *Emx2* mutant phenotype and patterns of *Emx2* expression suggest that it may also regulate regional patterning in this context. First, unlike other transcription factors required for hair cell development, EMX2 is not restricted to hair cells (Bermingham et al., 1999; Keithley et al., 1999; Wallis et al., 2003) and can be detected in non-sensory structures, including the transitional epithelial cells of the utricle, roof of the saccule and the endolymphatic duct. Second, in *Emx2* mutants the most prominent cochlear phenotype is not a reversal of stereociliary bundle orientation but rather a complete loss of OHCs coupled with severe IHC disorganization (Holley et al., 2010) (also Fig. 4B). Finally, as we have demonstrated, *Emx2* transcription initiates at a time when the otocyst is actively being patterned along its primary axes, which would be consistent with EMX2 contributing to these processes. Interestingly this stands in contrast to EMX2 function in the zebrafish lateral line, where EMX2 is expressed only in hair cells and expression does not initiate until after their specification. Moreover, in this system, *Emx2* is regulated by Notch signaling between nascent hair cell pairs so that EMX2 is expressed in only one cell and the pair develops with oppositely oriented stereociliary bundles (Jacobo et al., 2019; Kozak et al., 2020).

One possibility is that the primary function of EMX2 is to establish the regional identity of sensory epithelia, dividing the utricle and saccule into distinct functional domains with bundle orientation being the most salient anatomical demonstration that patterning has occurred. In this case, we predict that EMX2 would sit atop a gene regulatory network that patterns the utricle and saccule, and thus there are likely to be additional factors that coordinate EMX2 with GPR156 and STK32A. Alternatively, it is possible that EMX2 has multiple functions and that, after otocyst patterning, it acts in hair cells to regulate factors such as GPR156 and STK32A, which are required to orient the stereociliary bundle. This would be consistent with hair cell overexpression studies in which the EMX2 effect is direct and cell specific (Jiang et al., 2017). Distinguishing between these alternatives would reveal whether or not there are additional planar polarity factors waiting to be discovered or whether all the factors regulating vestibular hair cell polarization have been identified.

## MATERIALS AND METHODS

### Mouse lines and husbandry

All mice were housed at the University of Utah under Institutional Animal Care and Use Committee (IACUC) approved guidelines, and individual lines were maintained by backcross to B6129SF1/J hybrid females and genotyped using allele-specific PCRs (available upon request). For timed breeding and tissue staging, noon on the day in which a vaginal plug was seen was considered embryonic day 0.5 (E0.5), and the day litters were born was considered postnatal day 0 (P0). Mice from both sexes were used for experimentation. *Emx2*^Cre/WT^; *Rosa*^tdTomAi9/WT^ or *Emx2*^P2ACreERt2/WT^; *Rosa*^tdTomAi9/WT^ embryos were generated by crossing *Emx2*^Cre/WT^, *Emx2*^P2ACreERt2/WT^ or *Emx2*^P2ACreERt2/P2ACreERt2^ male mice with *Rosa*^tdTomAi9/tdTomAi9^ females, and staged and collected as described. *Emx2*^P2ACreERt2/P2ACreERt2^; *Rosa*^tdTomAi9/WT^ embryos were generated by crossing *Emx2*^P2ACreERt2/P2ACreERt2^ male and *Emx2*^P2ACreERt2/WT^; *Rosa*^tdTomAi9/WT^ female mice. *Emx2*^Cre/Cre^ embryos and *Emx2*^P2ACreERt2/P2ACreERt2^ pups were generated by intercrossing male and female heterozygotes for each allele. *Lmx1a Dreher* mutants were generated by intercrossing *Lmx1a*^Dre/WT^ males and females, whereas for *Emx2*-CreERt2 or *Emx2*-Cre lineage tracing, *Emx2*^P2ACreERt2/P2ACreERt2^; *Lmx1a*^Dre/WT^ or *Emx2*^Cre/WT^; *Lmx1a*^Dre/WT^ males were crossed with *Lmx1a*^Dre/WT^; *Rosa*^tdTomAi9/tdTomAi9^ females. *Emx2*-Cre mice (Kimura et al., 2005) were provided by S. Aizawa (Riken Institute, Wako, Japan). B6129SF1/J hybrid (strain 101043), *Lmx1a/Dreher* (strain 000636) and *Rosa* Ai9tdTOM (strain 007909) (Madisen et al., 2010) mice were purchased from The Jackson Laboratory.

### Emx2-CreERt2 mouse line production

A DNA sequence containing P2A and CreERt2 flanked by 800 bp of homologous DNA was inserted into the third exon of *Emx2* using the Easi-Crispr strategy (Quadros et al., 2017) developed in conjunction with the University of Utah Mutation Generation and Detection Core facility. This gene targeting strategy replaces the endogenous stop codon of *Emx2* with P2A, and was directed by a guide RNA (gRNA) targeting the genomic sequence ‘CCTCAGACGATTAAAAGTCCAAA’. Double-stranded donor DNA containing the P2A-CreERt2 construct and flanking homologous arms was co-injected with gRNA and Cas9 protein by the University of Utah Transgenic and Gene Targeting Core facility. Accurate homologous recombination was confirmed by PCR amplification across the 5′ and 3′ homologous arms using primers binding to the *CreERt2* sequence [Fig. 3, primer b (5′-CCGCCGCATAACCAGTGAAACAGC-3′) or primer c (5′-TGGCCCAGCTCCTCCTCATCCTCT-3′)] or the *Emx2* locus outside of either arm [Fig. 3, primer a (5′-GGACGGGAGCAGAGGAAAGAGACC-3′) or primer d (5′-CCATCCCAGTCCTGCTCCCTCATT-3′)]. Using this screening approach, 26% of progeny were identified that demonstrated *CreERt2* insertion at the *Emx2* allele. Three male founders (2, 15 and 45) with accurate homologous recombination within the 5′ and 3′ arms were further tested for Cre-recombinase activity resembling that of *Emx2*-Cre after tamoxifen induction at E15.5. A single male (2) was selected and backcrossed with B6129S2/J F1 hybrid female mice to establish the *Emx2-*CreERt2 transgenic line.

### Tamoxifen induction

For CreER-mediated lineage tracing experiments, CreERt2 was induced by a single dose of 3 mg tamoxifen (Sigma-Aldrich, T5648) and 3 mg progesterone (Sigma-Aldrich, P3972) prepared in corn oil (Sigma-Aldrich, C8267), and delivered via oral gavage to timed pregnant dams. Progesterone was included to reduce the likelihood of litter miscarriage due to tamoxifen toxicity. Pups were collected at P0 or gestational day 19.5 for dams that had not delivered litters before this stage.

### Immunofluorescent labeling and quantification

For whole-mount immunofluorescent labeling, heads were bisected and brains removed before immersion fixation of the intact inner ear and skull in 4% paraformaldehyde (PFA) solution (Electron Microscopy Sciences) prepared in 67 mM Sorensons phosphate buffer (pH 7.4). Tissue was dissected to isolate the cochlea and vestibular organs, and these were permeabilized for 30 min in blocking solution containing PBS with 1% bovine serum albumin (BSA) (Jackson ImmunoResearch 000-001-162) and 5% donkey serum (Jackson ImmunoResearch 017-000-121), supplemented with 0.5% Triton X-100 (Sigma-Aldrich T9284). Primary antibodies were diluted in blocking solution supplemented with 0.1% Tween-20 (Sigma-Aldrich 274348) and incubated with tissues overnight at 4°C. Tissue was rinsed thoroughly with PBS containing 0.05% Tween-20, and incubated overnight with species-specific AlexaFluor-conjugated secondary antibodies (Jackson ImmunoResearch 712-165-153, 705-545-147, 711-545-152, 711-605-152, 705-605-147, 711-165-152 and 715-165-150; all used at a 1:1500 dilution). Tissue was rinsed thoroughly with PBS containing 0.05% Tween-20 and mounted on slides using Prolong Gold (Thermo Fisher Scientific, P36930). For immunofluorescent labeling of cryosections, tissue was fixed with 4% PFA/Sorensons67 overnight at 4°C (P0 heads) or for 2 h on ice (embryos), then passed through a sucrose gradient and frozen in Neg-50 (Epredia). Sections were collected using a Leica CM3050 cryostat onto SuperFrost Plus slides (Fisher). Sections were labeled using a modified labeling protocol that excluded Tween-20 from the blocking and wash solutions. Fluorescent images were acquired by structured illumination microscopy using the Carl Zeiss Axio Imager M.2 with ApoTome.2 attachment and Axiocam 506 mono camera. Images were processed using Carl Zeiss Zen software, and figures prepared using Adobe Illustrator. FIJI (ImageJ) was used for quantification of tdTomato-labeled cells in in the sensory and non-sensory regions of the vestibular organs. For normalization of cell counts, the developmental stage with the greatest number of cells counted was assigned a value of 1 and the number of cells at that stage was used as the denominator to normalize cell counts at the remaining stages. Commercial antibodies used in this study were: mouse anti-βII Spectrin (BD Biosciences, 612562, 1:1000 dilution), rabbit anti-dsRed (Clontech/Takara, 632496, 1:5000 dilution), rabbit anti-MyosinVIIA (Proteus Biosciences, 25-6970, 1:800 dilution), goat anti-Oncomodulin (Santa Cruz Biotechnology, SC-7466, 1:250 dilution), goat anti-Prox1 (R&D Systems, AF2727, 1:300 dilution), goat anti-Sox2 (Santa Cruz Biotechnology, 17320, 1:200 for wholemount or 1:100 dilution for cryosection), rat anti-tdTomato (Kerafast, EST203, 1:500 for wholemount or 1:250 dilution for cryosection) and phalloidin Alexa Fluor 488 (Invitrogen, A12379, 1:1500 dilution). The rabbit anti-otolin antibody (1:1000 dilution) has been previously described (Deans et al., 2010) and was provided by G. Wong (Johns Hopkins University, Baltimore, MD, USA).

## Supplementary Material


