# Supplementary material for: Emx2 lineage tracing reveals antecedent patterns of planar polarity in the mouse inner ear
Source: Development. 2024 May 16;151(10):dev202425. doi: 10.1242/dev.202425 (PMC11165714; doi:10.1242/dev.202425)
Supplement: Supplementary file 1 [file develop-151-202425_review_history.pdf]

Emx2 lineage tracing reveals antecedent patterns of planar polarity in the mouse inner ear

Ellison J. Goodrich and Michael R. Deans
DOI: 10.1242/dev.202425

Editor: Francois Guillemot

Review timeline
Original submission: 12 October 2023
Editorial decision: 27 November 2023
First revision received: 11 April 2024
Accepted: 18 April 2024

Original submission

First decision letter

MS ID#: DEVELOP/2023/202425

MS TITLE: Emx2 Lineage Tracing Reveals Antecedent Patterns of Planar Polarity in the Mouse Inner Ear

AUTHORS: Ellison J Goodrich and Michael R Deans

I have now received the reports of two referees on your manuscript and I have reached a decision. The referees' comments are appended below, or you can access them online: please go to BenchPress and click on the 'Manuscripts with Decisions' queue in the Author Area.

As you will see, the referees express great interest in your work, but referee 2 in particular has significant criticisms and recommends a substantial revision of your manuscript before we can consider publication. If you are able to revise the manuscript along the lines suggested, which may involve further experiments, I will be happy to receive a revised version of the manuscript. I encourage you in particular to consider the experiments of early Emx2 lineage tracing and further investigation of Dreher mutants requested by referee 2. Your revised paper will be re-reviewed by one or more of the original referees, and acceptance of your manuscript will depend on your addressing satisfactorily the reviewers' major concerns. Please also note that Development will normally permit only one round of major revision. If it would be helpful, you are welcome to contact us to discuss your revision in greater detail. Please send us a point-by-point response indicating your plans for addressing the referees' comments, and we will look over this and provide further guidance.

Please attend to all of the reviewers' comments and ensure that you clearly highlight all changes made in the revised manuscript. Please avoid using 'Tracked changes' in Word files as these are lost in PDF conversion. I should be grateful if you would also provide a point-by-point response detailing how you have dealt with the points raised by the reviewers in the 'Response to Reviewers' box. If you do not agree with any of their criticisms or suggestions please explain clearly why this is so.

Reviewer 1

Advance summary and potential significance to field

© 2024. Published by The Company of Biologists under the terms of the Creative Commons Attribution License (https://creativecommons.org/licenses/by/4.0/).

This is a clear and well-illustrated paper that uses novel mouse tools to lineage trace Emx2-expressing progenitors in the mouse inner ear. Emx2 has been implicated in creating two zones of oppositely-polarized hair cells in the balance organs of the ear. The authors show that this asymmetry is present from the earliest times at which the balance organs start to differentiate. This raises the fascinating question of how polarity is specified so early.

Comments for the author

This is a lovely paper and I have no significant concerns.

Reviewer 2

Advance summary and potential significance to field

The study is well done but the advancement and significance is moderate.

Comments for the author

Emx2 is a transcription factor that mediates the line of polarity reversal (LPR) formation in the vestibular maculae of the inner ear. This study focuses on lineage tracing of Emx2 in the developing inner ear. The authors generated an Emx2 creER strain, which unlike the existing Emx2 cre line, does not disrupt endogenous Emx2 expression. Using temporal control of cre activation in the Emx2 creER strain, the authors demonstrated that the lateral region of the utricle and central region of the saccule where hair bundles are reversed from the default position, started to be labeled by Emx2 lineage at E11.5. These results suggest that the LPR is established early during development.

Additionally, the authors analyzed Emx2 lineage in the dreher mutants, in which the inner ear lacks morphogenesis and the sensory organs are often fused. The authors reported similar Emx2 lineage domain along one side of the fused utricle and saccule. Taken together, the authors concluded that the LPR is established early by the expression of Emx2 within the prosensory domain.

Overall, the manuscript is clearly written, and the figures are clearly illustrated. While the results of Emx2+ domains at P0 being labeled by Emx2 lineage at E11.5 are clear, it is not clear how the overlap of Emx2+ and Sox2+ domains at the medio-posterior otocyst at E11.5 (Fig. 2) evolves into the lateral domain of the utricle and the center region of the saccule (postulated in the Discussion). It would be helpful to conduct some shorter-term harvests such as E12 or E12.5, after TM injection at E11.5 to follow the Emx2 lineage domains.

Furthermore, it is debatable whether the results of the dreher strengthen the authors' hypothesis because the mutant inner ear is so malformed that it is hard to correlate the Emx2 lineage in the prosensory domain of WT at E11.5 (Figure 2F and G) with the lineage domain shown in Figure 8. I think investigating hair bundle orientation in the dreher mutants is important to lend support to the notion that Emx2 lineage also overlaps with the sensory domain in the dreher mutants.

Results of the two suggested experiments above will help to strengthen the authors' hypothesis and may also help the authors to hypothesis the different hair bundle patterns observed between the utricle and saccule.

Other suggestions
1) Add normalization of labeled cells to the Method section.
2) Abstract, line 5, it is ok to describe Emx2 expressed in one side of the utricle, but it is not expressed in one side but rather in the center region of the saccule.
3) Figure 5, add dates of TM injection on the utricle panels.
4) Figure 7, add A, B, C and D to panels. "Transitional epithelia" should be "transitional epithelial cells".
5) Figure 7, last panel. It is not clear why the data points for hair cell and support cell curves were not aligned.
6) Sentence above the subtitle of "Emx2 lineage tracing throughout inner ear development", remove one of the "in"s.

© 2024. Published by The Company of Biologists under the terms of the Creative Commons Attribution License (https://creativecommons.org/licenses/by/4.0/).

7) In the “Emx2 lineage tracing throughout inner ear development” paragraph, line 8, should read, saccule, and anterior and horizontal “cristae”, not “canals”.
8) 4th paragraph of the same section, it seems like proliferation could play a role in more labeled supporting cells and transitional epithelial cells at a later age. In contrast, I don’t see how greater Emx2 expression in supporting cells and transitional epithelial cells could account for the increase in lineage+ cells at later ages, considering the reporter activity is driven by the Rosa and not Emx2 promoter upon cre-mediated recombination. Please clarify.

First revision

Author response to reviewers' comments

(1) While the results of Emx2+ domains at P0 being labeled by Emx2 lineage at E11.5 are clear, it is not clear how the overlap of Emx2+ and Sox2+ domains at the medio-posterior otocyst at E11.5 (Fig. 2) evolves into the lateral domain of the utricle and the center region of the saccule (postulated in the Discussion). It would be helpful to conduct some shorter-term harvests such as E12 or E12.5, after TM injection at E11.5 to follow the Emx2 lineage domains.

Author Response: We agree that tracking the event(s) that separate the saccular epithelia from the prosensory domain and utricle, and following Emx2-Cre labeling during these events could clarify our understanding of the morphogenesis of these structures. Unfortunately, we have not been able to reconstruct this 3-dimensional process satisfactorily in 2-dimensional sections and foresee the need for 3D reconstruction using imaging techniques that are beyond our current capabilities. Nonetheless we agree that it is of great interest to know more about the Emx2+, Sox2+ domain. Towards this end we have evaluated ears at E12.0 using antibodies against the otoconial membrane protein OTOLIN and are able so demonstrate that cells within this region of overlap express this marker that also defines the mature maculae. This result is consistent with this region evolving into the utricle and saccule and has been added to a restructured Figure2.

(2) I think investigating hair bundle orientation in the dreher mutants is important to lend support to the notion that Emx2 lineage also overlaps with the sensory domain in the dreher mutants.

Author Response: To accomplish this, we bred mice that allowed us to conducted Emx2-Cre lineage tracing on the Dreher mutant background and looked at stereociliary bundle orientation along the Emx2-lineage boundary. Emx2- Cre was selected for these experiments because it is more efficient than Emx2-CreERt2 and therefore allowed us to evaluate stereociliary bundle orientation in all cells along the boundary. As shown in an updated Figure 8, immunofluorescent labeling of SPECTRIN shows hair cells within the Dreher mutant sensory domain that overlap with the Emx2-lineage, and reveals bundle orientations from hair cells located on either side of the Emx2-lineage boundary. One region has an organization that clearly resembles the LPR of the utricle and another has characteristics that are consistent with the saccule though lacking the precise organization of a wild type control. We appreciate the recommendation and agree that this addition improves the manuscript.

…

Other suggestions:

1) Add normalization of labeled cells to the Method section.

Thank you. This step has been added to the Methods.

2) Abstract, line 5, it is ok to describe Emx2 expressed in one side of the utricle, but it is not

© 2024. Published by The Company of Biologists under the terms of the Creative Commons Attribution License (https://creativecommons.org/licenses/by/4.0/).

expressed in one side but rather in the center region of the saccule.

The abstract has been edited to meet the 180 word limit and this point has been clarified

3) Figure 5, add dates of TM injection on the utricle panels.

This oversight has been corrected

4) Figure 7, add A, B, C and D to panels. “Transitional epithelia” should be “transitional epithelial cells”.

This oversight has been corrected

5) Figure 7, last panel. It is not clear why the data points for hair cell and support cell curves were not aligned.

Thank you, this formatting error has been corrected

6) Sentence above the subtitle of “Emx2 lineage tracing throughout inner ear development”, remove one of the “in”s.

Thank you

7) In the “Emx2 lineage tracing throughout inner ear development” paragraph, line 8, should read, saccule, and anterior and horizontal “cristae”, not “canals”.

Thank you

8) 4th paragraph of the same section, it seems like proliferation could play a role in more labeled supporting cells and transitional epithelial cells at a later age. In contrast, I don’t see how greater Emx2 expression in supporting cells and transitional epithelial cells could account for the increase in lineage+ cells at later ages, considering the reporter activity is driven by the Rosa and not Emx2 promoter upon cre-mediated recombination. Please clarify.

Author Response: Our rationale is that, when the dose of tamoxifen remains constant, the probability of an individual cell being labeled is dependent upon the amount of CreERt2 that cell is expressing at the time of tamoxifen induction. Based upon this we expect that cells with higher levels of CreERt2 expression would be labeled with a greater frequency than cells with low levels of expression. However, this reasoning did not consider cellular events occurring after tamoxifen induction including proliferation. Therefore, we agree that the data does not exclude proliferation as a contributing factor and we have clarified the text accordingly. More importantly we have also added the statement that ‘these recombination profiles will be important to consider if the Emx2-CreERt2 line is to be used for targeted gene deletion and the production of conditional knockout mice’.

Second decision letter

MS ID#: DEVELOP/2023/202425

MS TITLE: Emx2 Lineage Tracing Reveals Antecedent Patterns of Planar Polarity in the Mouse Inner Ear

AUTHORS: Ellison J Goodrich and Michael R Deans
ARTICLE TYPE: Research Article

I am delighted to tell you that your manuscript has been accepted for publication in Development, pending our standard ethics checks.

© 2024. Published by The Company of Biologists under the terms of the Creative Commons Attribution License (https://creativecommons.org/licenses/by/4.0/).

Reviewer 2

Advance summary and potential significance to field

Emx2 is a transcription factor that mediates the line of polarity reversal (LPR) formation in the vestibular maculae of the inner ear. Using lineage tracing and the dreher mutants, the authors demonstrated that Emx2 mediates this function early, prior to sensory organ formation.

Comments for the author

The authors have made a good faith effort in strengthening the hypothesis that the transcription factor Emx2 establishes hair bundle orientation in the developing prosensory domain. The revised manuscript is acceptable for publication.

© 2024. Published by The Company of Biologists under the terms of the Creative Commons Attribution License (https://creativecommons.org/licenses/by/4.0/).
